# Comparative Analysis of Gait Speed Estimation Using Wideband and Narrowband Radars, Thermal Camera, and Motion Tracking Suit Technologies

**DOI:** 10.1007/s41666-020-00071-7

**Published:** 2020-04-16

**Authors:** P. P. Morita, A. S. Rocha, G. Shaker, D. Lee, J. Wei, B. Fong, A. Thatte, A. Karimi, L. Xu, A. Ma, A. Wong, J. Boger

**Affiliations:** 1 School of Public Health and Health Systems, University of Waterloo, Waterloo, ON Canada; 2 Centre for Global eHealth Innovation, Techna Institute, University Health Network, Toronto, ON Canada; 3 Institute of Health Policy, Management, and Evaluation, University of Toronto, Toronto, ON Canada; 4 Research Institute for Aging, Waterloo, ON Canada; 5 Goiano Federal Institute, Trindade, GO Brazil; 6 Department of Electrical and Computer Engineering, University of Waterloo, Waterloo, ON Canada; 7 Department of Mechanical and Mechatronics Engineering, University of Waterloo, Waterloo, ON Canada; 8 Waterloo Artificial Intelligence Institute, Waterloo, ON Canada; 9 Department of Systems Design Engineering, University of Waterloo, Waterloo, ON Canada

**Keywords:** Gait speed detection, Wideband radar 10 GHz, Narrowband radar 24 GHz, Thermal camera, Motion detection suit, Comparative analysis

## Abstract

Research has shown that cognitive and physical functioning of older adults can be reflected in indicators such as walking speed. While changes in cognition, mobility, or health cause changes in gait speed, often gradual variations in walking speed go undetected until severe problems arise. Discrete clinical assessments during clinical consultations often fail to detect changes in day-to-day walking speeds and do not reflect walking speeds in everyday environments, where most of the mobility issues happen. In this paper, we compare four walking speed measurement technologies to a GAITRite mat (gold standard): (1) an ultra wideband radar (covering the band from 3.3 GHz to 10 GHz), (2) a narrow band 24-GHz radar (with a bandwidth of 250 MHz), (3) a perception Neuron Motion Tracking suit, and (4) a thermal camera. Data were collected in parallel using all sensors at the same time for 10 healthy adults for normal and slow walking paces. A comparison of the sensors indicates better performance at lower gait speeds, with offsets (when compared to GAITRite) between 0.1 and 20% for the ultra wideband radar, 1.9 and 17% for the narrowband radar, 0.1 and 38% for the thermal camera, and 1.7 and 38% for the suit. This paper supports the potential of unobtrusive radar-based sensors and thermal camera technologies for ambient autonomous gait speed monitoring for contextual, privacy-preserving monitoring of participants in the community.

## Introduction

Previous research has shown gait (walking) speed to be a valuable and reliable indicator for the assessment of an individual’s health, particularly mobility and cognitive status [13, 36]. People with age-related conditions, such as Alzheimer’s disease and frailty, often present changes in gait speed that are challenging or impossible to monitor during clinical visits [11]. Currently, a gait speed assessment is only advised for those who have already been diagnosed with relevant issues and have been referred to a physician or hospital [34]. Variations in gait speed as a result of cognitive or other conditions may go undetected as the effect is gradual and often not noticeable during clinic visits. Ubiquitous and unobtrusive technologies that detect changes in gait speed of older adults in clinical (long-term care, hospitals) and non-clinical environments (retirement homes, independent living, etc.) could support detection, evaluation, and monitoring of parameters related to changes in mobility, cognition, and frailty. As shown by previous studies (e.g., [18, 34]), the ability to recognize and handle such conditions is critical for supporting health and quality of life for older adults, especially for those who choose to live independently.

### Wearable-Based Gait Monitoring

The most common technologies used to monitor gait speed are wearable based, such as accelerators and gyroscopes [26], or operate in a controlled test environment, such as the GAITRite mat [7]. The GAITRite mat is a pressure sensor–based mat that is able to provide accurate real-time data regarding multiple aspects of an individual’s walking characteristics, such as cadence, walking speed, right and left step and stride lengths, and right and left step times [25]. GAITRite has been used extensively in research for comparison against other methods that attempt to accurately measure speed and other important gait metrics. Studies that examined gait metrics for older adults and post-stroke patients had reliable results in detecting spatio-temporal gait parameters under single- and dual-task conditions [6]. The GAITRite mat has also been used for measurement of temporal and spatial gait parameters, in agreement with a separate method of calculation involving footfall count and stopwatch timing [43]. The GAITRite mat is compatible with other gait-monitoring technologies (e.g., Vicon [27]) and can be used in conjunction with other systems to act as the gold-standard in validating new systems [25].

In previous research, one study used three-axis accelerometer-based sensors to measure the kinematics of separate body segments with possible medical applications when calculating gait parameters, although no estimates of gait speed were provided [22]. Bamberg et al. [3] investigated the efficacy of portable wearable sensors installed in participants’ shoes to model gait outside of the confines of a lab environment and reported an average error of 6.5 ± 11.7% for the stride length. Another study overcame the challenge caused by joint angles and lack of flat surfaces on the human body to investigate the placement of inertial measurement unit (IMU) sensors on selected joint ends and muscle extensions and its effect in gait analysis [32]. While wearables are relatively cheap to manufacture and are portable, some disadvantages limit their uptake such as challenges in setup, compliance, and maintenance, particularly for older adults who have physical or cognitive limitations as they would need to remember and commit to wearing and charging the devices regularly.

Other strategies to measure gait speed have used smartphones or tablets embedded with Global Positioning System (GPS)[26, 33]. Although these devices are more practical and popular, they lack accuracy when tracking subtle gait speed changes and are only capable of monitoring when the person is carrying them. An in-home method using Kinect depth cameras has been introduced for gait speed monitoring [12, 35], but it is not suitable to cover large fields of view and the use of computer vision usually creates privacy concerns for the end users.

Most of the technologies discussed in this section provide accurate methods for gait speed monitoring but have limited applicability in the field for the monitoring of gait speed in contextual settings as seniors’ residences and in independent living settings. Privacy in video-based tracking systems and adherence in wearable-based systems are the major limitations in the update of these technologies for in situ, contextual studies.

### Wireless Gait Monitoring

In an effort to solve privacy and practical issues, researchers have been working on alternative wireless radar technologies to monitor walking speed. For example, (quasi-)zero-effort ambient gait speed tracking systems with considerable low privacy constraints have been developed. Geisheimer et al. [14] focused in a descriptive motion method and modeling with potential application in clinical gait analysis and biometric identification, but authors did not report the accuracy of their technology. Saho et al. [31] compared groups of younger and older adults with a principal component analysis to identify correlation between speed and fall risk, but did not compare the results to ground truth so were not able to report on accuracy or error.

Different types of low-frequency (5–8 GHz) radars for gait speed assessment have been reported using a variety of continuous wave (CW) and pulse Doppler radars [9, 40]. Ciddihy et al. measured gait velocity and compared them to the ones taken by a clinician with a stopwatch. With some radar angle adjustments and a calibration factor, the average reported error was 10.5%. Wang et al. compared their speed results to a Vicon system as ground truth, with the speed estimated by the radar to be 87% the speed estimated by the Vicon. In previous research, we investigated radar-based walking speed measurement using a 24-GHz radar that achieved an accuracy of 90.5% versus ground truth data from a GAITRite mat at slower walking speeds [5]. With a greater focus on gait analysis, other researchers have proposed a system that uses a frequency-modulated continuous wave (FMCW) radar to measure not only walking speed but also stride length [16]. With an ultra wideband radar, researchers were also able to mitigate wall interference when detecting motion and have explored the classification of different objects and body parts [37]. This research can be used in the future towards differentiating between different people in the same area and even separate an individual’s legs as two entities in an effort to calculate stride length. With this type of radar, the system may also be accurate at longer distances, as determined by its tracking range [42].

The radar system’s granularity for recognizing distinct gait speeds is a crucial factor when considering clinical applications, as the system needs to accurately detect subtle variations over time. In this regard, the radar’s frequency range is an important feature. Our previous research investigated two types of radar: low frequency (5–10 GHz) and higher frequency (24 GHz), where we measured the efficacy for slow, normal, and fast gait speeds for healthy younger adults using a pre-built tracking algorithm [4, 20]. While the radars were useful for distinguishing between different gait speeds for the younger adults, all the walking speeds were faster than would be seen in a frail older adult, with maximum average accuracy of approximately 86% at fast speeds, 81% for the normal, and 74% for the low speeds. For these types of sensors to be used to detect clinically significant changes in health, they must be able to accurately detect walking speeds comparable with frail older adults.

Gait estimation has also been explored using infrared thermal cameras. In one study, Lu et al. [21] proposed the use of thermal imaging techniques to estimate gait using a link model based on human body joints and a support vector machine (SVM) classifier to extract a feature vector of each joint. Kim et al. leveraged a model-based object tracking algorithm with a robust silhouette extraction (accuracy superior to 90% when compared with manually inputted ground truth) with potential application for gait monitoring [19]. DeCann et al. designed a complex gait monitoring system based on the detection of silhouette from thermal images and demonstrated a significant improvement in estimation accuracy [10].

## Objectives

The objective of this work is to perform a comparison of privacy-preserving technologies that could be used to detect normal and frail walking speeds. Four sensing technologies were examined: (1) a 3–10 GHz ultra wideband radar–based sensor, (2) a 24-GHz narrowband radar–based sensor, (3) infrared thermal camera, and (4) motion detection suit. All sensor technologies were compared to a GAITRite mat as the gold standard used to represent a ground truth system.

The sensors included in this study were selected based on (a) their ability to present user privacy, (b) the non-obtrusive nature of these sensors, and (c) the ease of use and setup in contextual studies in a naturalistic setting. Radar-based sensors provide a complete privacy-preserving, non-obtrusive solution that can be potentially mounted behind walls; infrared thermal cameras provide an alternative to vision-based systems that can preserve user privacy; and the motion detection suit can be used in naturalistic settings for a more in-depth analysis of gait while allowing participants to move freely through their home environment.

This manuscript provides the health informatics community with insights about (a) the accuracy of these systems, (b) limitations of these technologies for studies in naturalistic settings, and (c) guidance on the use of these technologies for specific types of studies.

## Methodology

The development of a platform able to identify when a person’s maximum gait speed has fallen below a clinical threshold requires technologies that can monitor people in their home. The radar-based deployments and the thermal camera tested in this work provide a non-intrusive method for detecting older adults’ gait speed. However, it must be established whether or not the detection level is granular enough for a clinician to determine whether the person being monitored requires a more detailed clinical assessment. In that sense, the technology must accurately detect walking speed variations, especially when they fall below 0.65–0.7m/s, which has been determined to be the clinical threshold indicator of frailty [1, 24]. Furthermore, to the best of our knowledge, no previous work has compared the use of radar-based gait estimation methods along with thermal camera and a motion detection suit to detect such low gait speed ranges.

We used commercially available sensors for this study, with customized analytics to extract the gait speed indicators presented in this manuscript. While the sensory technology is available in the market, the two radar-based solutions and the infrared thermal camera solution were implemented with customized software developed by our research team at the University of Waterloo. The selected technologies present a novel privacy-preserving method for monitoring gait speeds in naturalistic settings through a combination of sensors and customized analytics software developed for our platform. This is also the first time that these sensors have been compared to each other on a single study, providing insights on how they perform compared to other privacy-preserving technologies.

### Study Protocol

**Fig. 1:**
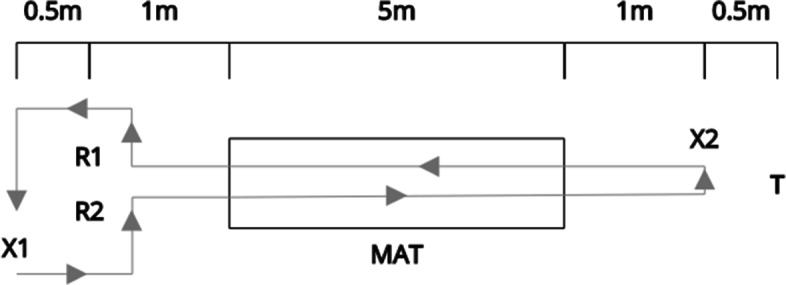
Schema for one round trip with two walks. R1 and R2 are the 10-GHz and 24-GHz radars. T is the thermal camera, and X1 and X2 are the starting and ending points respectively

As our previous work determined no difference in simulated gait speed for older and younger adults [5], gait was captured from ten healthy young adults, with an average age of 21 (*σ* = 1.8); 50% males and 50% females; and with an average self-reported weight of 65 Kg (*σ* = 11.1); and self-reported height of 1.68 m (*σ* = 0.1). Participants consisted of a convenience sample of ten university students that self-identified as healthy individuals with no gait impairments as our goal was to compare different gait estimation technologies without normal gait patterns. The sample size used in this study is comparable to previously conducted pilot studies [8]. Each participant was asked to take part in two trials, one walking at a normal speed ($\sim $1.0 m/s) and a second one at a slower speed ($\sim $0.6 m/s). Each trial included two intervals split by a break ($\sim $5 min). Each trial consisted of six walks (3 there-and-back round trips; see Fig. 1).

Before the first walking interval, the participant was asked to put on the motion tracking suit with the help of its operator and run through a series of calibration postures and movements. Once finished calibrating the suit, the participant went through a trial walkthrough on the mat of the walking procedure with one of the operators.

During each walking interval, the participant started from a spot located 1 m behind the two radars, walked 7 m continuously in a straight line in front of the radars along the GAITRite. Both starting and turning points were marked on the floor with a tape. The turning point was placed approximately 1 m after the end of the mat, such that acceleration and deceleration effects are attenuated. The GAITRite system also requires the participant to walk off the mat in order to split each walk and there is a 1-m buffer area in front of the beginning of the mat to prevent the radar pulses blurring out when the participants walk too close to the radars. Prior to completing the second trial (slow walking pace), the researchers demonstrated the target walking speed and asked rate with a metronome [41] playing at 60 bpm.

### Equipment Setup

#### GAITRite

**Fig. 2:**
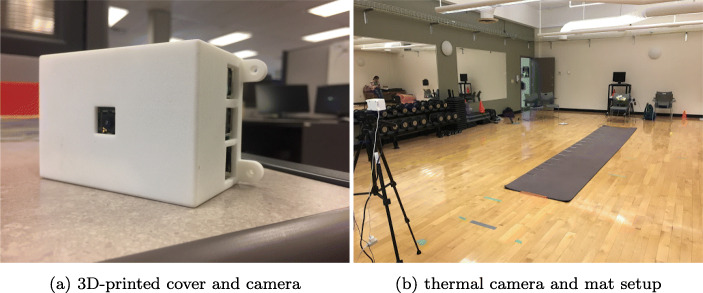
Equipment setup

The GAITRite mat was set up in the center of a spacious room to avoid obstacles and possible noise from the surrounding environment as well as to provide a clear field of vision for the sensors. The mat was placed on a flat surface to produce clear footprints in the data capture system to calculate gait metrics without any warps. The computer system responsible for data capture and the power supply for the sensors were plugged in the front of the mat, behind the 10-GHz and 24-GHz radars, as shown in Fig. 2b. The 14’ model of the GAITRite mat is composed of over 16,000 touch sensors that are placed throughout its extension, which measures out at uncut factory dimensions of 90 cm × 5.2 m (35” × 204”) with a 6 mm height for the electronics box. The active portion of the mat where sensors record data measures out at 61 cm × 4.27 m. The system uses a sampling rate of 60 Hz and higher with a high spatial accuracy (± 1.27 cm). Measured based on a suspended walk format, the mat detects each footfall and outputs a footprint as the first part of data collection. After manual verification by the operator, it is confirmed that each of the 6 walks is split properly and each footprint is recorded and validated. The GAITRite mat system then adds each walk together for a total of 12 walks per participant. Its physical contact with the participant’s foot and kinematic measurements based on these recorded footprints and elapsed time make this system very accurate in calculating walking speed and other gait metrics.

#### 10-GHz Radar

The 3–10-GHz ultra wideband radar–based system used in this study is a commercial solution manufactured by Vayyar [38]. This device has a frequency range of 3–10 GHz and employs an 18 antenna element linearly polarized broadband array. The system comes with open application programming interfaces (APIs) that provide raw signal data which can be used for development purposes [39], and which were used in the development of our customized analytics tools.

#### 24-GHz Radar

The second radar-based system employed a commercial radar sensor (IMST SENTIRE^*TM*^ Radar Module) that operates in continuous wave (CW) mode in the frequency range of 24 GHz [17]. Doppler effect provides information regarding the velocity of a target in the received signal. In our scenario, when a person is walking towards the radar, it will induce an increase in the frequency of the received signal, and vice versa. Therefore, walking speeds can be estimated by extracting the frequency shifts in the radar signal. In this study, we employ a short-time Fourier transform (STFT) to calculate the spectrogram of the received radar signal and visualize the frequency changes induced by the walking person. STFT is applied according to the below formula:

1 $$ S(t,f)=\left | \sum\limits^{\infty}_{n=-\infty} w(n)x(t-n)e^{-j2\pi fn} \right |^{2}  $$

After obtaining the spectrogram, we then extracted the points with the highest intensity values and average the frequency shifts represented by these points. The averaged frequency shift is then converted to be the walking speed. Both radars were attached to a stand at a height of approximately 2 m off the ground. This gives the best result for single body reflection for the radar pulses when a participant walks on the mat.

#### Thermal Camera

The thermal camera setup used a Raspberry Pi [30] to extract frames from the sensor, using the pylepton open source library [15]. The infrared ray from the human body is transformed into an electric signal and the FLIR (forward-looking infrared) thermal camera captures a sequence of thermal images as the target is walking towards and away from the setup. By analyzing the thermal characteristics of the images, the gait rate of the target can be approximated by reading the pixel intensity values of the thermal frames. A custom-made enclosure was designed and 3D-printed to house the FLIR camera and Raspberry Pi, as shown in Fig. 2a. The enclosure was mounted on a standard tripod for the camera to reach a broader field of view. As seen in Fig. 2b, this tripod had a height of 1.5 m and was placed 1–2 m away from the end of the GAITRite mat, extending out along the end of the mat. This setup allowed for a full upper body capture of the subject throughout the test, while also capturing enough data from the subject when they stood the furthest away. This arrangement captured the thermal and geometrical characteristics of the people while they were walking between the near end and the far end of the camera. The camera recorded about 13 frames per second and a total of 1000 frames for each participant in each test, adding up to 20k frames for the entire experiment. The thermal camera essentially captures the temperature magnitude and the size of the target contained in number of pixels occupied by the target, both of which are related to the distance between the camera and the target. By extracting these features for each image frame, we can infer the location and the path of the target. The sum of the bright intensity of all pixels in one frame characterizes the overall magnitude of the temperature of the target. The features of all frames are then plotted as a time series, which helps to identify frames with key locations during the movements that reveal the path patterns of the target. Since we know the time each frame is recorded, the speed of the target can be estimated by relating the distance traveled during the key locations to the time interval of the associated frames.

#### Motion Tracking Suit

**Fig. 3:**
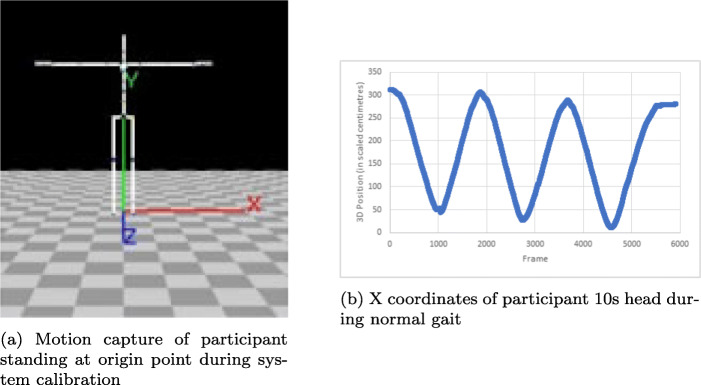
Motion tracking suit

This study investigated the performance of the Perception Neuron Accelerometer Suit (PNAS) [29] in estimating gait speed wirelessly using accelerometers. The suit was used to collect gait-related data, which was then processed post-capture to determine velocity relative to the portions of walking that corresponded to the GAITRite mat. PNAS is able to capture full-body kinematics using individual sensors called “neurons” that attach on to different parts of the suit and contain an in-built inertial measurement unit (IMU) with a gyroscope, accelerometer, and magnetometer. The suit can connect wirelessly to the Axis Neuron software [28] to provide real-time data on user motion. Data output includes a Biovision Hierarchical data (BHD) file showing the motion capture animation of the user (Fig. 3a), as well as quantitative data as joint acceleration and position of all sensors. The suit is composed of straps for each limb segment, as well as the head and torso. The system is set at a standard frame rate of 125 frames per second and provides various kinematic measures including acceleration and position. To extract the walking speed, the 3D coordinates of the participant’s head were graphed, as shown in Fig. 3b, and minimum/maximum inflection points in the graph of the X and Y coordinates were used to determine overall displacement for each lap. This displacement was divided by the time for each lap (dividing number of frames in a lap by the frame rate) to find overall displacement.

## Results

This manuscript focuses on reporting the comparative performance and the discussion of advantages and benefits for the health informatics community of four non-obtrusive, privacy-preserving wireless technologies for monitoring gait speed: two radar-based sensors, one infrared thermal camera sensor, and one motion suit. In the results section, we present the results of our pilot study.

### GAITRite

**Fig. 4:**
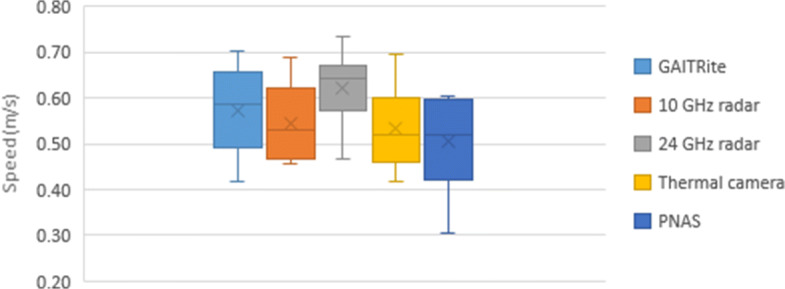
Slow speed comparison

**Fig. 5:**
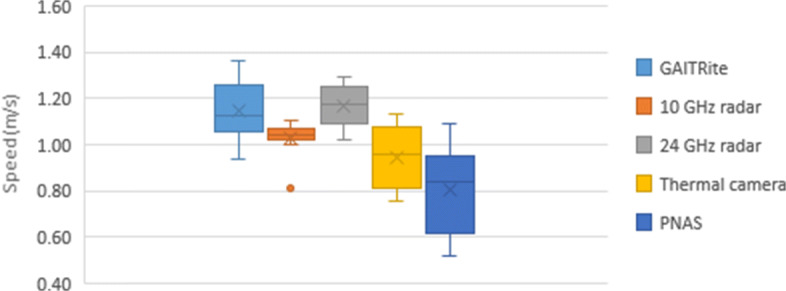
Normal-speed comparison

The data from the GAITRite mat was processed by identifying each step in a time series and using each step as a metric for the next. In order to calculate the distance between steps, it determines the distance traveled from the heel of the first footstep to the toe of the last footstep. After the first footfall, the system triggers an internal timer that stops after the last footstep is recorded or when the participant’s foot leaves the mat. The system uses the distance-time ratio to calculate speed per segment over the traveled area on the mat. The actual speed used for comparison against other systems being tested in this study is calculated by averaging all six walks together for each trial (normal or slow speed). If a walk is considered abnormal, particularly when the footstep lands too lightly on the mat, an alternative method is used. This light step leads to a soft footprint that is not recorded properly, skewing the data, as it results in an abnormally low or high speed (halved or doubled from the average), or produces an error when trying to compute the related walk. On such rare occasions, the average of the speeds would omit this walk and use an average of 5 walks rather than 6. These issues were detected in participants 1, 2, and 3 for one normal walking. The final average speed and corresponding standard deviation for each participant when measured using the GAITRite mat are presented in Table 1. The speed distributions are presented in Fig. 4 for slow pace and Fig. 5 for normal pace.

**Table 1:** Average gait speed (m/s) for each participant for the five gait speed estimation technologies

Participant	Pace	GAITRite	Radar 10 GHz	Radar 24 GHz	FLIR	PNAS
1	Normal	1.06	1.02	0.84	0.81	0.62
	Slow	0.42	0.46	0.47	0.42	0.43
2	Normal	1.09	1.08	0.87	0.81	0.78
	Slow	0.70	0.69	0.68	0.63	0.59
3	Normal	1.03	1.00	0.93	0.75	0.52
	Slow	0.62	0.58	0.66	0.55	0.52
4	Normal	1.09	1.05	0.91	0.93	0.90
	Slow	0.66	0.62	0.67	0.59	0.60
5	Normal	1.29	1.07	1.11	1.14	1.02
	Slow	0.66	0.63	0.73	0.70	0.60
6	Normal	1.36	1.11	1.20	1.08	1.09
	Slow	0.64	0.56	0.66	0.55	0.60
7	Normal	0.94	0.81	0.90	0.81	0.69
	Slow	0.56	0.50	0.63	0.49	0.30
8	Normal	1.20	1.03	1.08	0.98	0.60
	Slow	0.53	0.49	0.58	0.49	0.52
9	Normal	1.25	1.07	1.22	1.09	0.93
	Slow	0.43	0.46	0.56	0.45	0.40
10	Normal	1.17	1.02	1.02	1.02	0.92
	Slow	0.51	0.47	0.57	0.46	0.49
$\bar {x}$	Normal	1.15	1.03	1.01	0.94	0.81
	Slow	0.57	0.55	0.62	0.53	0.51
*σ*	Normal	0.13	0.08	0.14	0.14	0.19
	Slow	0.10	0.08	0.08	0.09	0.10

### Wideband Radar

**Fig. 6:**
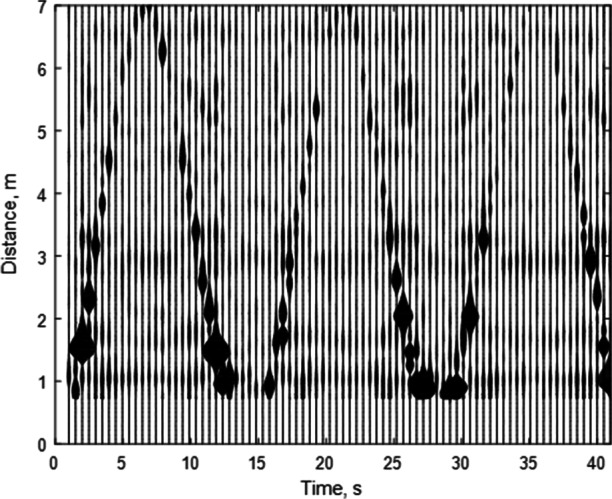
Radar data collected from Walabot (10 GHz) after data processing

In order to collect the data, we ran a Python script from a laptop (with Python version 3.4 and Walabot API installed) using the Anaconda Command Prompt [2]. The script initiated the radar, collecting background data for a few seconds to be used in clutter noise removal before prompting the user to run a participant. Post-processing was used to subtract the background data (which should not contain any movement) from the actual data, filtering out the noise from the surroundings. Then, the relevant part of the signal values fit into a graph plotting distance of the target with the number of pulses sent, which progresses in value over time. Figure 6 illustrates the trajectory of the moving target, where the further away the target is from the radar, the weaker the signal is for the reflected pulses. Speed was estimated using distance and time elapsed for each lap. Post-processing and walking speed estimation were performed using MATLAB scripts [23], which were custom built to process data from the excel files and produce a graph of the results. The final average speed and corresponding standard deviation for each participant are presented in Table 1. The speed distributions are presented in Fig. 4 for slow pace and Fig. 5 for normal pace.

The wideband of 6–8-GHz radar was used for tracking the walking human in two different cases: slow and fast mode. The radar can transmit a temporally short pulse and recode the echo pulse with one-dimensional range information. To keep track of the trajectory of every walking response, the one-dimensional range profile was stacked to visualize the whole procedure. The speed of walking was estimated using two pieces of information: the stacked range profile where it is based on real-time measurement and the required time (elapsed time) for light to travel each relative distance. Each subject walked away from and towards the radar six times with three turnarounds, and the six-speed information was calculated per every measurement. The average speed of each mode of walking was obtained by taking the average of all the measurements related to that mode.

### Narrowband Radar

**Fig. 7:**
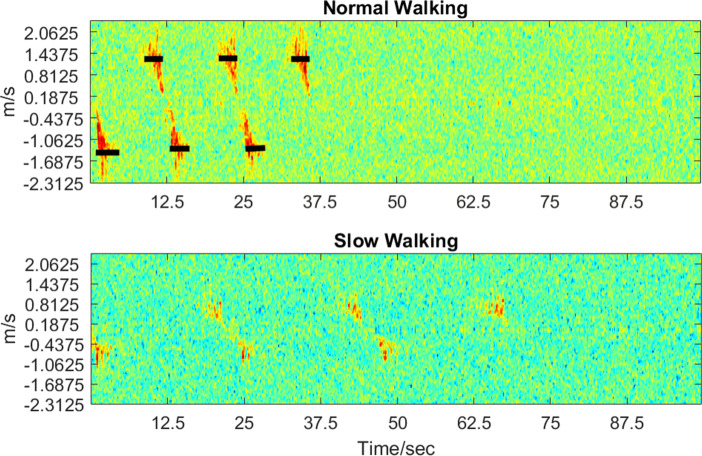
Spectrograms showing the difference in normal walking and slow walking speeds for 24-GHz radar. The three upper marks show average positive speeds and three lower marks show average negative speeds, with positive speed representing the person walking towards the radar and negative speed representing the person moving away from the radar

The 24-GHz radar operates in a similar approach to the 10-GHz radar, where the radar is used for range detection of a moving subject. Fast Fourier Transforms on the range data enable velocity/speed determination. Given the different frequencies of operation, along with different achievable bandwidths, each of the radars would have different capabilities in detection and clutter removal. The 24 GHz produced the spectrograms shown in Fig. 7, where the six markers represent the six walking segments. As seen in Fig. 7, the speed expansion (the red area) in the normal speed spectrogram is much larger than that of the slow speed spectrogram. The negative marks in the normal-speed (top) spectrogram indicate an acceleration period at the start of the segment, followed by a plateau (constant speed), then a blank area as the participant left the radar’s field of view. As for the positive marks, we see a steady plateau as the participant enters the radar’s field of view, followed by a deceleration period. The spectrogram representing slow walking speeds shows smaller marks with no apparent speed variations. With these marks, we then extracted the points with highest intensity values and averaged the frequency shifts represented by these points. The averaged frequency shift *Δf*, the base frequency *f*_0_, and the light speed *c* are used to calculate the walking speed *Δv* with the Doppler shift formula (2). The final average speed and corresponding standard deviation for each participant are presented in Table 1. The speed distributions are presented in Fig. 4 for slow pace and Fig. 5 for normal pace.

2 $$ {\varDelta} v=c\frac{{\varDelta} f }{f_{0}}  $$

### Thermal Camera

**Fig. 8:**
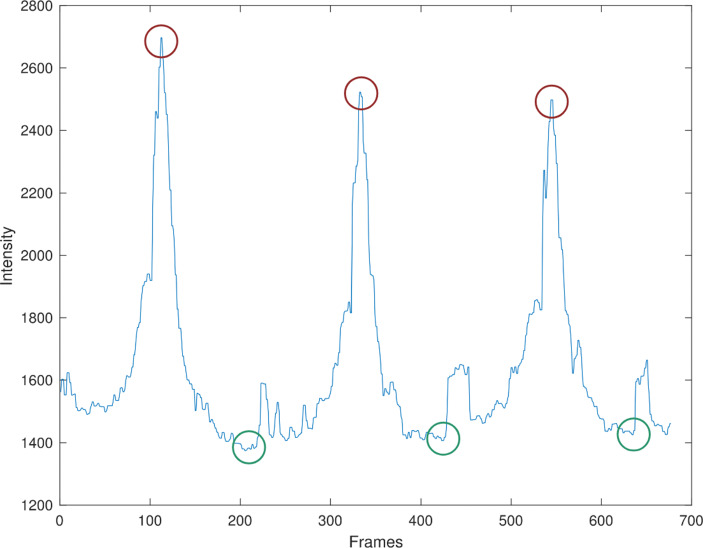
Sum of pixel intensity for captured thermal frames: participant 7, normal speed

**Fig. 9:**
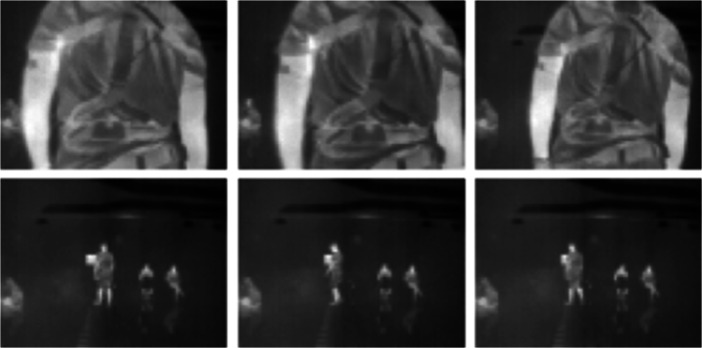
Example thermal captures made using the thermal camera

As mentioned in Section 3, when a participant approaches the thermal camera, the number of pixels occupied by the target increases in the acquired images. Instead of capturing the thermal characteristics of the background, the newly occupied pixels are now measuring the temperature of the participant. As such, the sum of pixel intensity values reaches a maximum value when the target is closest to the thermal camera. Figure 8 illustrates the sum of intensity values over all pixels for each captured thermal frames of participant 7, when performing normal walking speed. We can observe three distinct local maxima (red circles at peaks) that correspond to the three times when the participant was right in front of the thermal camera. The three green circles (valleys) indicate when the participant is positioned at the farthest distance from the thermal camera. The top three pictures of Fig. 9 show the frames associated with the peaks mentioned above. All three thermal frames showcase the instant in which the participant finished walking towards the sensor and started walking away from the camera. The same observation can be made using the three local minima presented in Fig. 8: the three extracted thermal frames (bottom three pictures of Fig. 9) correspond to the local minima and indicate the moment when the participant reached the furthest distance from the camera. The final average speed and corresponding standard deviation for each participant are presented in Table 1. The speed distributions are presented in Fig. 4 for slow pace and Fig. 5 for normal pace.

A further observation of Fig. 9 indicates that the local minima are not as easily identifiable since the ambient thermal background together with the non-target object can profoundly affect the overall pixel intensity values. Therefore, the local maxima are leveraged as critical locations to help identify the distance of the target from the setup. As the thermal camera is capturing frames at a constant frame-per-second rate, the duration between each local maxima can be estimated using the number of frames between each peak and the camera’s capture rate. Since the participant travels a fixed distance between the key locations, the speed of the participant can be estimated with a simple $\frac {\text {distance}}{\text {time}}$ ratio.

### Motion Tracking Suit

The PNAS walking speed estimation used 3D graphed coordinates along with minimum/maximum inflection points to determine overall displacement for each walk. This displacement was divided by the time for each walk (calculated diving number of frames in a walk by the frame rate) to generate six total speed measurements. These were averaged to determine the speed of each participant under both the fast and slow conditions, as shown in Table 1 along with standard deviation. The speed distributions are presented in Fig. 4 for slow and Fig. 5 for normal walks.

### Summary of Results

On average, the technologies we tested deviated between 7.3 and 10.5% when compared to the GAITRite for slow speed measurements. In regard to normal speeds, the deviation ranged between 10.1 and 30%; the FLIR and PNAS had greater deviations at normal speed. Table 2 summarizes the errors, also presenting the values in meters per second for the sensor type.

**Table 2:** Average errors and standard deviations when compared to GAITRite

Error		Radar 10 GHz		Radar 24 GHz		FLIR		PNAS	
Unit		Slow	Normal	Slow	Normal	Slow	Normal	Slow	Normal
%	$\bar {x}$	7.33	10.11	10.33	12.29	9.48	18.04	10.54	30.08
	*σ*	2.99	6.59	8.53	6.16	4.53	5.8	11.61	12.48
m/s	$\bar {x}$	0.04	0.12	0.05	0.14	0.05	0.21	0.07	0.34
	*σ*	0.02	0.09	0.04	0.07	0.03	0.06	0.07	0.13

### Secondary Analysis

**Fig. 10:**
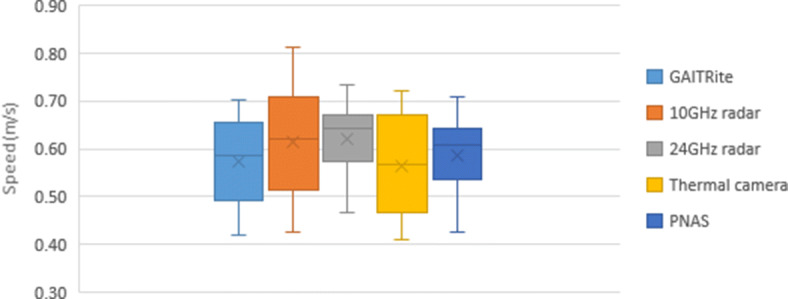
Slow speed: secondary analysis for GAITRite-equivalent portion of data

**Fig. 11:**
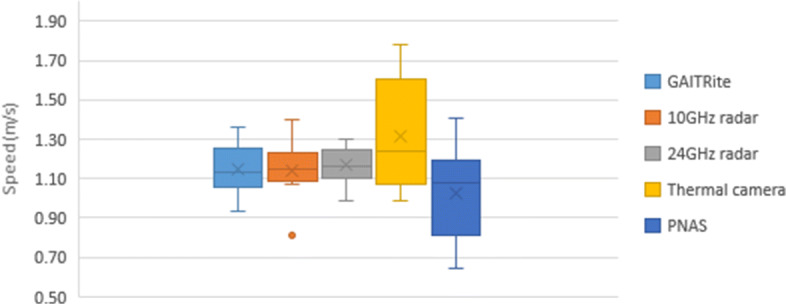
Normal speed: secondary analysis for GAITRite-equivalent portion of data

For the $\sim $7 m for each walk, only 5 m was traversed over the GAITRite mat. Therefore, only the middle 5 m of walk were captured by the mat while the other systems collected data for the entire walk, including acceleration and deceleration that were not recorded by the mat. As the aim of this study is to compare the gait monitoring capabilities of the proposed systems to the GAITRite mat as the gold standard; a secondary analysis was conducted to control for the acceleration and deceleration periods at the beginning and end of the round trip. In other words, to compare only the portion of data from the sensors that aligned with data captured by the GAITRite mat rather than the entire walk. To achieve this, we cropped the sensor data to isolate the middle interval that aligned with the data collected from the GAITRite mat. The final GAITRite-equivalent average speed and corresponding standard deviation for each participant and each sensor are presented in Table 3. The speed distributions are presented in Fig. 10 for slow pace and Fig. 11 for normal pace.

**Table 3:** Average gait speed (m/s) for each participant for the five gait speed estimation technologies using the secondary analysis that compared data for the GAITRite-equivalent portion of the data

Participant	Pace	GAITRite	Radar 10 GHz	Radar 24 GHz	FLIR	PNAS
1	Normal	1.06	1.23	0.99	1.08	0.82
	Slow	0.42	0.42	0.47	0.41	0.44
2	Normal	1.09	1.40	1.11	1.11	1.02
	Slow	0.70	0.81	0.68	0.71	0.68
3	Normal	1.03	1.16	1.10	1.04	0.64
	Slow	0.62	0.62	0.66	0.60	0.59
4	Normal	1.09	1.13	1.13	1.18	1.13
	Slow	0.66	0.68	0.67	0.66	0.71
5	Normal	1.29	1.24	1.23	1.78	1.21
	Slow	0.66	0.79	0.73	0.72	0.63
6	Normal	1.36	1.08	1.27	1.61	1.41
	Slow	0.64	0.64	0.66	0.60	0.62
7	Normal	0.94	0.81	1.10	0.99	0.92
	Slow	0.56	0.63	0.63	0.51	0.57
8	Normal	1.20	1.09	1.24	1.30	0.79
	Slow	0.53	0.54	0.58	0.54	0.62
9	Normal	1.25	1.09	1.30	1.61	1.16
	Slow	0.43	0.47	0.56	0.42	0.43
10	Normal	1.17	1.16	1.20	1.45	1.19
	Slow	0.51	0.53	0.57	0.48	0.57
$\bar {x}$	Normal	1.15	1.14	1.17	1.31	1.03
	Slow	0.57	0.61	0.62	0.56	0.59
*σ*	Normal	0.13	0.15	0.10	0.28	0.23
	Slow	0.10	0.13	0.08	0.11	0.09

#### 10-GHz Radar

To simplify the velocity calculation for the GAITRIte portion of the data, we have invoked the time-frequency (pulsed-based) dual nature of the radar system. The technology used in this study is a pulsed radar where the system produces a pulse repetitively within a specific time gap. This means that as the number of pulses sent increases, the elapsed time also increases. We scaled the total time in each type of walk to map against the total number of pulses originally used to convert the axis units. This conversion enables a straight-forward speed calculation $\left (\frac {\text {distance}}{\text {time}}\right )$ for each segment of the Fig. 6 by taking only the middle section of each walk over the mat. Participants traversed the mat at a constant speed (as verified by our research team), with the deceleration and acceleration portions happening off the mat. Then, all six segments were averaged together for each participant and particular interval (normal or slow walk) and compared to the gold standard. For slow speed, the average error of the 10-GHz radar dropped from 7.3 to 6.8% while for normal speeds the average error increased from 10.11 to 12.05%.

#### 24-GHz Radar

The traditional deployment of the 24-GHz radar specifies a speed resolution of 6.5 m/s [17]. However, we have shown that it is possible to use micro-doppler radar signal processing to probe further in resolution [5]. However, this approach comes with a limitation in the usable field of view, which was found to be around 3.5 m range in our trials. As the radar was located 1 m away from the GAITRite mat, the data captured when the participant was walking on the mat was approximately 2.5 m. As the participants were walking at a relatively constant speed on the mat, we selected data points marked by the black bars in Fig. 7 for a secondary analysis, which isolates constant walking speed for the participant from accelerating/decelerating data. As seen in Table 4 and Fig. 11, this secondary analysis makes the normal-speed estimation more accurate, dropping the average deviation from 12.3 to 5.8%. For slow speeds, Fig. 7 shows that there are no obvious “constant speed” regions, making the acceleration/deceleration periods much shorter. This means it is not possible to separate data points that represent the constant speed when the participant is actually walking on the mat.

**Table 4:** Average errors and standard deviations when compared to GAITRite portion of the data in the secondary analysis

Error		10-GHz radar		24-GHz radar		FLIR		PNAS	
Unit		Slow	Normal	Slow	Normal	Slow	Normal	Slow	Normal
%	$\bar {x}$	6.83	12.05	10.33	5.80	3.52	13.51	5.09	12.51
	*σ*	7.24	8.41	8.53	4.41	3.04	13.15	4.40	13.95
*m/s	$\bar {x}$	0.04	0.14	0.05	0.06	0.02	0.17	0.03	0.14
	*σ*	0.05	0.10	0.04	0.04	0.02	0.17	0.03	0.15

#### Thermal Camera

As the first pre-processing step of the analysis, the captured segments in which the participant was walking off the mat when starting each walk and when making a 180-degree turnaround are removed from the total duration of the thermal data. The primary reason for this pre-processing step is that, since acceleration and deceleration were involved in the action of a turnaround, using the entire duration of the thermal data would not accurately reflect the gait rate. Therefore, it is necessary to remove these segments to enable consistent gait speed estimation. Furthermore, the exact time interval when each participant was on the mat was not recorded during the experiment. To account for this, the gait rate estimation using thermal data is based on the assumption that it took approximately 2 s after the participant reaches the end of each walk to perform the turnaround and resume walking on the mat. More specifically, the computational method for estimating gait speed can be described as follows. First, the local maxima and minima with respect to the sum of pixel intensities of the individual frames are identified, and the time duration of the walk is calculated using their corresponding frame indices. Next, to approximate the interval when participant was walking on the mat, we subtract 4 s from the total time. Finally, the speed is estimated by dividing the length of the mat using time. This approach reduced the average deviation for slow speeds from 9.4 to 3.5%, and from 18 to 13% for normal speeds.

#### Motion Tracking Suit

In the secondary analysis for the PNAS, 250 frames (equivalent to 2 s of data) were used to find the velocity of the participant for each walk. The speed and error was calculated in the same way as for the primary analysis. Comparing the calculated error shown in Tables 2 and 4, there is a significant drop in the average deviation from 10.5 to 5% for slow speeds and from 30 to 12.5% for normal speeds. This is likely because while the participant walked out of the field of view for some portion of the other sensors, the PNAS captured the entirety of all walks, including turnaround motions, which introduced greater sources of perceived error.

## Discussion

When comparing Figs. 4, 10, 5, and 11, as well as Tables 2 and 3, it appears there is difference in the accuracy of the speed calculations. These differences are likely occurring because the GAITRite is recording steady-state walking speed from the middle of the walk. As the other sensors recorded most or all of the participants’ walk, including acceleration, deceleration, and turnaround, it is expected that the sensors would demonstrate a lower performance compared to GAITRite when considering the whole walk. After the secondary analysis that focused just on data that aligned with the GAITRite-equivalent portion of participants’ walk, we noted an average error lower than 10.3% for all technologies when estimating slow speeds. For the normal pace, the maximum average error reached 13.5%. Specifically for the 24-GHz radar, the experiment was limited by the short detection range of the radar and the adopted micro-doppler scheme. Future versions of the technology could address this issue and potentially show better accuracy with the same experiment set up. These results also emphasize (a) the importance of having a minimal distance between the individual and the sensors to prevent blurring and (b) that the accuracy of these systems is higher for steady speed measurement than for the acceleration and deceleration cycles. Therefore, it is important for innovators using this technology to consider these limitations when using these technologies. Radar-based sensors would work well for the measurement of walking speed along a hallway, but would have limited accuracy to monitor the variations in speed immediately after a person leaves the bed.

Through the analysis of thermal camera data, we found that the magnitude of the intensity values and the number of bright pixels representing the target vary significantly with distance changes between the target and the camera. By extracting features for each frame to reflect the intensity and size characteristics, we identified some critical locations during the movement based on the time series pattern of the extracted features over all frames. With that, we infer the speed of the movement based on the distance and the time interval between key locations. However, we perceived that, on average, the speed estimation from thermal frames slightly underestimates for 7 out of the 10 slow-pace experiments when compared to the GAITRite results. It is important to realize that the theoretical limitation of the thermal camera speed measurement is determined by its own frame rate. That is, given a 15-frame-per-second capture rate and a walking speed of 1 m/s, the measurement provided by the thermal camera can be most precise to 0.07 m/s. With additional information, deep learning–driven approaches could be leveraged to autonomously extract distinct features from the thermal frames, constructing a more accurate frame displacement model.

While using the PNAS, the data accuracy varies considerably in comparison with the ground truth GAITRite mat when considering the entire 7-m path for each walk. Part of the inaccuracy could be a result of calibration difficulties due to switched leg sensors for some participants. This setup anomaly may indicate incorrect data, resulting in skewed speeds for these trials. Besides that, where speed was calculated accurately, the PNAS tended to result in a lower speed than that yielded by the GAITRite Mat. As the PNAS was the only sensor that was never “out of range,” greater discrepancies could be a result from deceleration, acceleration, and turn-around periods off the mat. This speculation is supported by the drop in error when we ran the secondary analysis considering only the GAITRite-equivalent of each walk.

Considering the secondary analysis with better results regarding the deviations from the GAITRite and comparing to previous research cited in the introduction of this paper, we see a potential opportunity to apply wireless gait monitoring technologies to gait speed in home settings for monitoring steady speeds. Our study reached average errors between 6.8 and 10.3%, particularly for slow speed estimation. The referenced studies reported accuracy between 80 and 90%, when compared to other gold standard methods. This is comparable or better than values reported by other research groups for other monitoring technologies.

When doing an experiment to compare different systems, one of the biggest challenges is synchronization. While the sensor systems have timestamping abilities, all the five systems (including GAITRite) operate independently. A secondary analysis that selects valid data periods based on global synchronization is therefore difficult to implement. Additionally, the four systems tested in this study have their own strengths and shortcomings in detecting gait speed, which impact the optimal timeframe for gait detection. This resulted in differences in the suitable valid data period corresponding to optimal performance of different systems. Thus, while the detection accuracy of each system was not calculated based on exactly the same data, the validity of system performance is comparable for optimal data capture for each system type. As the intention is to assess these systems for real-world application, the gait speed detection algorithm should be optimized based on the features and capabilities of each different device.

In addition, taking into account the various processes which are required to start the data capture for each independent system, it becomes difficult to gauge valid periods for analysis when each system’s strength lies in its own method and timeframe for data collection. Since each method relies upon different inputs and methods for initialization, it becomes harder to control the synchronization, where it would be helpful instead to focus on studying the impact of being able to correlate results taken in relatively the same timeframe. To encompass each individual system’s differences, buffer time was placed at the beginning to aid in the synchronization of each test run. However, based on the goals of the study, having each system accurately capture data and then using the results to compare against other systems within the same timeframe are likewise suitable ways to determine the validity of the different devices in the best approach to confronting synchronization.

Overall, the radar-based sensors showed potential for further analysis in real-world monitoring applications due to their ease of setup and privacy-assuring approach for data collection. Radars have shown the most promise in the design of a solution that enables contextual, in situ monitoring of gait speed and variations, while preserving user privacy and being a non-obtrusive solution. Particularly, the ultra wideband radar had an average deviation of only 6.8% when compared to GAITRite. While the thermal camera also showed little deviation from the mat, it requires complex processing and could create privacy concerns as it is still possible to identify silhouettes in the thermal imaging. This poses some concerns of in-home use in private residences, but still provides a solid method for monitoring gait speeds in public settings as the common areas of retirement homes. The PNAS was the most complex system to setup, tune, and process with the greatest variation in speed distributions. While complex to use, the PNAS would be an ideal solution for naturalistic studies in which gait speed needs to be measured in multiple different locations. The scope of this study, in its first stage, was to estimate gait speed and future work will explore how this method compares to video or marker-based systems. Our results demonstrate that these technologies are all sensitive to acceleration/deceleration, providing an opportunity implement other applications, such as fall detection.

## Conclusion

This study presents a comparative analysis of unobtrusive sensor-based technologies that might be used for in-home walking speed monitoring. While this study is exploratory in nature, the results indicate that radar- and thermal-based systems could be used for monitoring gait as they can detect walking speeds that includes levels used as a clinical indication of frailty, while providing fairly accurate estimates of gait speed. Future research should examine the applicability of the suggested systems in real home environments as well as use with multiple people. The technologies reviewed in this paper provide ideal tools for naturalistic monitoring of gait speed, allowing patients to be monitored outside of the controlled environment of a medical clinic. Technologies as described in this paper have the potential to prevent falls, monitor the deterioration of physical and cognitive state, and empower seniors to live independently in the community.
